# Pneumonia due to *Pandoraea Apista* after evacuation of traumatic intracranial hematomas:a case report and literature review

**DOI:** 10.1186/s12879-019-4420-6

**Published:** 2019-10-22

**Authors:** Chuanzhong Lin, Ning Luo, Qiang Xu, Jianjun Zhang, Mengting Cai, Guanhao Zheng, Ping Yang

**Affiliations:** 1 Department of Pharmacy, Huadu District People’s Hospital of Guangzhou, Southern Medical University, Guangzhou, China; 2 Department of Pharmacy, The First Affiliated Hospital of Medicine School, Zhejiang University, Hangzhou, China; 3 Department of Pharmacy, Zhejiang provincial hospital of TCM, Hangzhou, China; 4 Department of Pharmacy, Meizhou People’s Hospital, Meizhou, China; 5 Department of Pharmacy, Shenzhen Hospital of Southern Medical University, Shenzhen, China

**Keywords:** *Pandoraea Apista*, Brain trauma, Pneumonia, Pathogenicity, Susceptibility

## Abstract

**Background:**

*Pandoraea* species is a newly described genus, which is multidrug resistant and difficult to identify. Clinical isolates are mostly cultured from cystic fibrosis (CF) patients. CF is a rare disease in China, which makes *Pandoraea* a total stranger to Chinese physicians. *Pandoraea* genus is reported as an emerging pathogen in CF patients in most cases. However, there are few pieces of evidence that confirm *Pandoraea* can be more virulent in non-CF patients. The pathogenicity of *Pandoraea* genus is poorly understood, as well as its treatment. The incidence of *Pandoraea* induced infection in non-CF patients may be underestimated and it’s important to identify and understand these organisms.

**Case presentation:**

We report a 44-years-old man who suffered from pneumonia and died eventually. Before his condition deteriorated, a Gram-negative bacilli was cultured from his sputum and identified as *Pandoraea Apista* by matrix-assisted laser desorption ionization–time-of-flight mass spectrometry (MALDI-TOF MS).

**Conclusion:**

*Pandoraea* spp. is an emerging opportunistic pathogen. The incidences of *Pandoraea* related infection in non-CF patients may be underestimated due to the difficulty of identification. All strains of *Pandoraea* show multi-drug resistance and highly variable susceptibility. To better treatment, species-level identification and antibiotic susceptibility test are necessary.

## Background

*Pandoraea* species were first described by Coenye et al. in 2000 [1], which had been isolated from both environmental and human clinical samples, mostly from cystic fibrosis (CF) patients. They may contribute to the lung function decline in CF patients. Some of these organisms are capable of causing bacteremia in both CF and non-CF patients. And yet, with limited evidence presented, the pathogenicity of *Pandoraea* remains poorly understood. Besides, it is difficult to differentiate *Pandoraea* species from some other species, such as *Burkholderia* or *Ralstonia* [2]. Furthermore, these bacteria are resistant to a lot of antibiotics, which makes the treatment of *Pandoraea* related infections more complicated. *Pandoraea* is rarely found in non-CF patients, due to different lung environments and misidentification. We report a case of pneumonia caused by *Pandoraea Apista* after the evacuation of traumatic intracranial hematomas and review the available literature concerning *Pandoraea* species to a better understanding.

## Case presentation

A 44-year-old man was transferred to the emergency intensive care unit (EICU) of The First Affiliated Hospital of Zhejiang University, Hangzhou, China, on November 25, 2018, due to multiple injuries and coma after a brain injury. Five days earlier, he accidentally fell from a height of about 7 m and immediately fell into a coma. Removal of traumatic intracranial hematoma and decompressive craniectomy were performed on November 20, 2018, and November 24, 2018. And antimicrobial treatment had been given before he was admitted to our hospital.

**Fig. 1:**
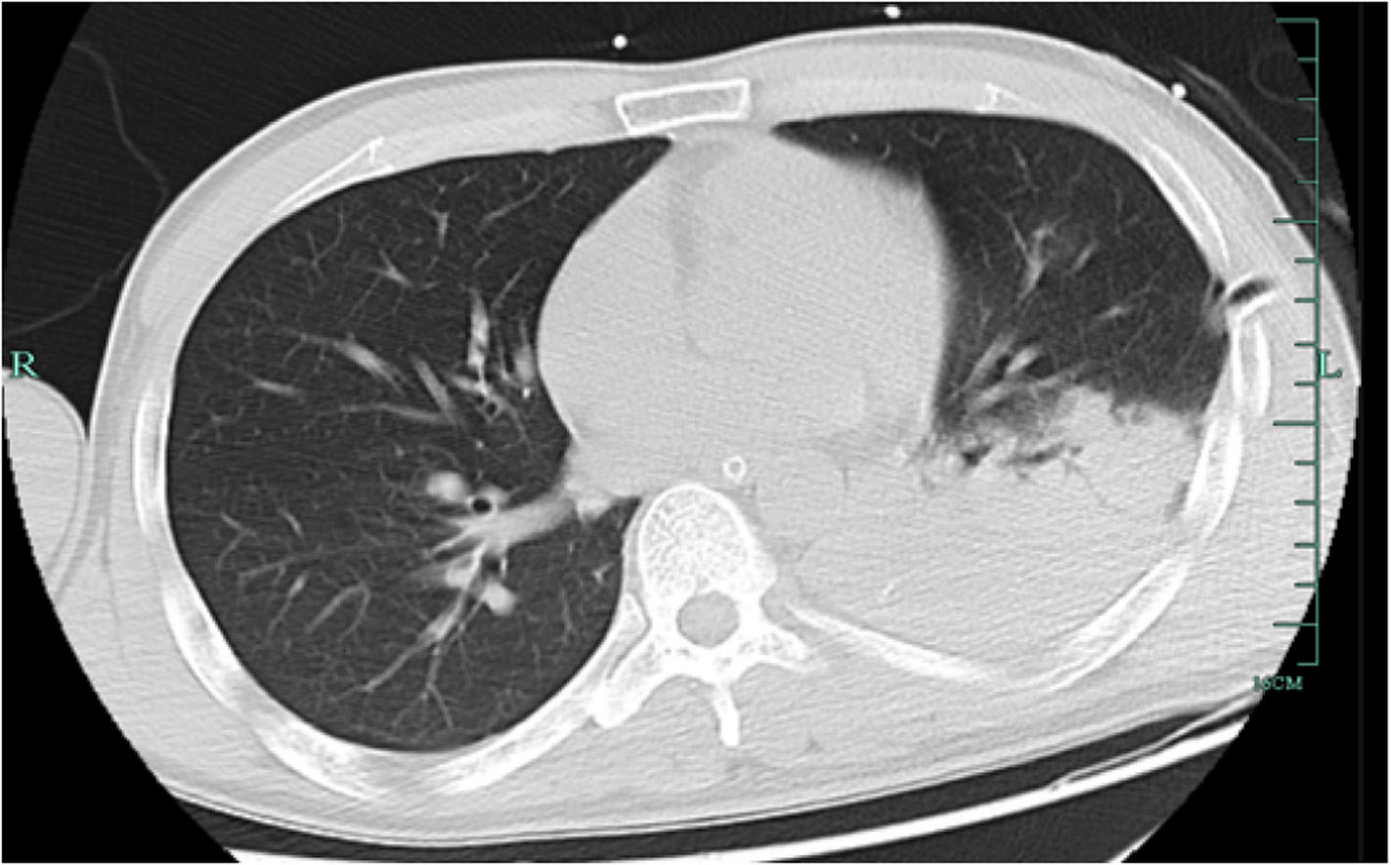
Lung CT scan on November 27, 2018. Lung CT taken on day 3 shows patchy consolidation in left inferior lobar

**Fig. 2:**
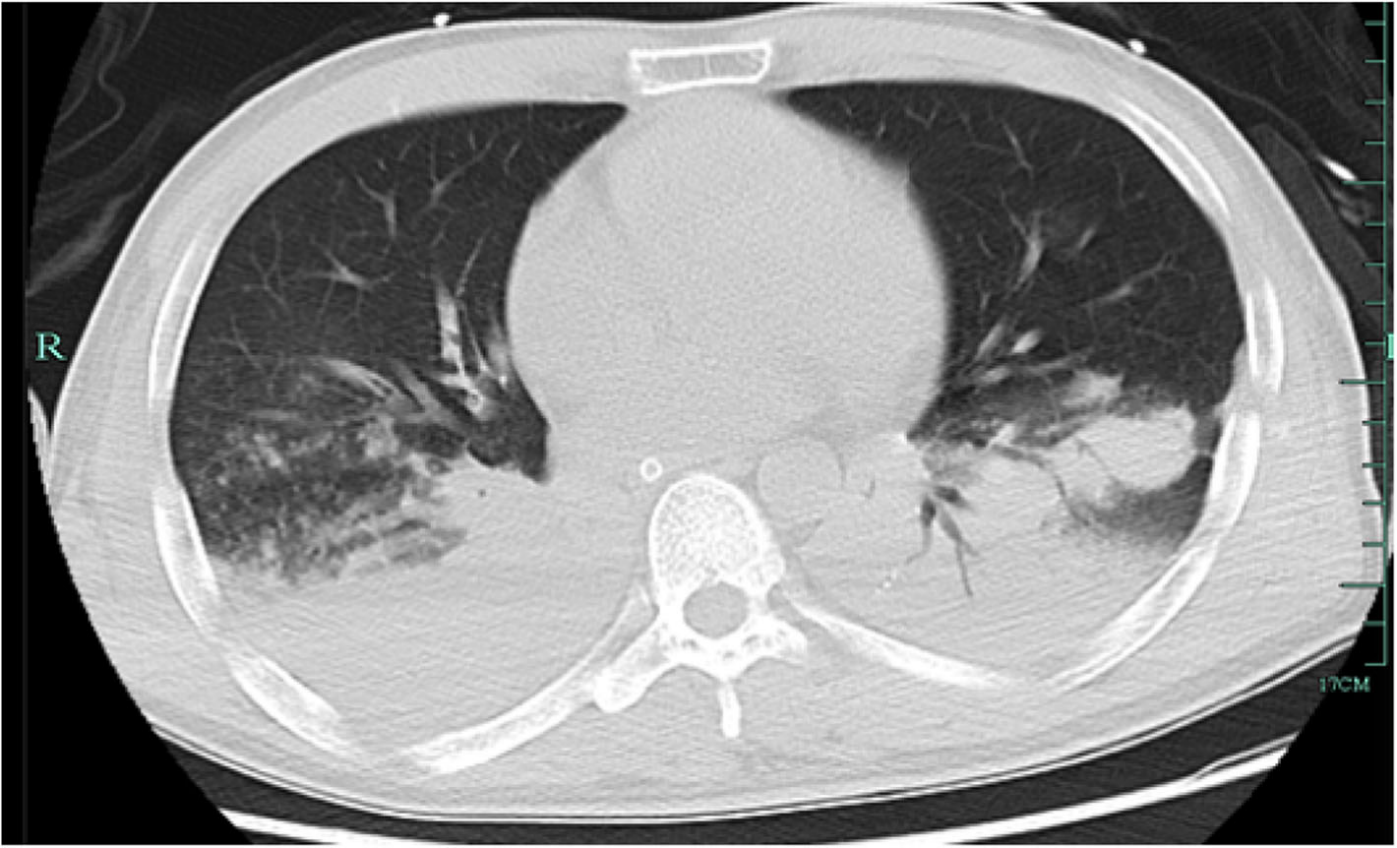
Lung CT scan on January 4, 2019. Lung CT taken on day 11 shows patchy consolidation in both lungs

On the day of admission, physical examination showed a low-grade fever of 37.6 °C, a blood pressure of 165/79 mmHg and a Glasgow Coma Score of 1 + T + 1. Laboratory examination detected an elevated white blood cell count (17.1 × 10E9/L with 90.6% neutrophils) and hypersensitive C-reactive protein (hsCRP) of 209.70 mg/L. Procalcitonin (PCT) was 0.38 ng/ml in the meantime. With tracheal intubation and ventilator-assisted ventilation were given, blood gas values were as follows: pO_2_, 117 mmHg; pCO_2_, 31.4 mmHg. After two sets of blood culture were taken, an antibiotic regimen included meropenem (2 g IV, 8 hourly) and vancomycin (1million IU IV 12 hourly) was given. A lung computed tomography (CT) scan was performed on day 3 (Fig. 1) and found patchy consolidation in left inferior lobar. It was considered as traumatic wet lung and/or lung infection. On day 7, blood culture showed no bacteria growth, the hsCRP decreased to 6.7 mg/L, PCT was 0.12 ng/ml, but the white blood cells were still elevated (12.3 × 10E9/L with 89.5% neutrophils). A large amount of Gram-negative rod from the sputum specimen taken at day 5, which identified as *Pandoraea Apista* by MALDI-TOF MS, was reported at day 8, with no antimicrobial susceptibility test results. The antibiotic regimen remained unchanged because of lack of knowledge about this genus and the infection of this patient seemed to be getting better. However, hsCRP and PCT increased progressively after that. It became more and more difficult to maintain his blood pressure and oxygen saturation. On day 11, the hsCRP was 194.4 mg/L. CT scan (Fig. 2) confirmed new infections in his right lung. The sputum culture result was reconsidered, and the microbiologist of our hospital confirmed that *Pandoraea Apista* was the only germ grow in the media. After a review of some case report concerning *Pandoraea* species, meropenem was altered by imipenem (1 g IV, 6 hourly) on day 12. Nonetheless, his condition got worse and the relatives of him asked for a “Do Not Attempt Resuscitation”. He died on day 14 with cardiac respiratory arrest and multiple organ failure.

## Discussion and conclusion

The timeline of our case-patient is summarized in Table 1. *Pandoraea* species is a strange genus to both clinicians and microbiologists in China. It is reasonable to believe that *Pandoraea apista* might not the responsible pathogen at the time of admission. But the using of broad-spectrum antibiotics promoted the growth of *Pandoraea apista*, which lead to the right pneumonia and a worsening condition of this patient. To better understand *Pandoraea* species and its pathogenicity, we present a review from literature reported before November 31, 2018.

**Table 1:** Case-patient timeline

Dates	Relevant Past Medical History and Interventions		
	No particular medical history		
Dates	Summaries from Initial and Follow-up Visits	Diagnostic Testing	Interventions
Day 1	5 days history of muti-injury caused by high falling; coma; status after removal of traumatic intracranial hematoma and decompressive craniectomy; fever	Body temperature: 37.6 °C; Blood pressure: 165/79 mmHg; Glasgow Coma Score: 1 + T + 1; White blood cell count: 17.1 × 10E9/L; Neutrophils%: 90.6%; hsCRP: 209.70 mg/L; PCT: 0.38 ng/ml; pO_2_: 117 mmHg; pCO_2_: 31.4 mmHg; X bedside photography: Exudative changes in the left lung, left rib fractures.	Antibiotic regimen: meropenem 2 g IV, 8 hourly and vancomycin 1million IU IV 12 hourly; Symptomatic treatment
Day 3	Left lung infection	Body temperature: 38.2 °C; Cranial plain CT: Changes after craniocerebral surgery, multiple intracranial hemorrhages, subarachnoid hemorrhage; Lung CT plain scan: Patchy consolidation in left inferior lobar, left rib fractures	
Day 7	Patient got better after treatment	Body temperature: 37.4 °C; White blood cell count: 12.3 × 10E9/L; Neutrophils%: 89.5%; hsCRP: 6.7 mg/L; PCT: 0.12 ng/ml; Blood culture: No bacteria growth after 7 days’ culture	
Day 8	*Pandoraea Apista* was considered as a colonization	Sputum culture: *Pandoraea Apista* (identified by MALDI-TOF MS)	
Day 11	Infection in both lungs; new confirmed infection in right lung	Body temperature: 38.0 °C; White blood cell count: 20.5 × 10E9/L; Neutrophils%: 95.2%; hsCRP: 194.4 mg/L; Lung CT plain scan: Patchy consolidation in both lungs	
Day 12	Physicians got the information from the literature that *Pandoraea Apista* may be resistant to meropenem but sensitive to imipenem	Body temperature: 39.2 °C; White blood cell count:22.1 × 10E9/L; Neutrophils%: 93.6%; hsCRP: 260.7 mg/L; pO_2_: 67.2 mmHg; pCO_2_: 39.2 mmHg;	Antibiotic regimen changed to imipenem 1 g IV, 6 hourly and vancomycin 1million IU IV 12 hourly
Day 14	Patient died		

### Microbiology, distribution, and identification

*Pandoraea* species belongs to the β-subclass of the *Proteobacteria*, which contains a group of Gram-negative bacilli that are aerobic or facultative anaerobic (e.g. *P. pnomenusa* reported by Ambrose et al. [3]), do not form spores, do not reduce nitrate, do not ferment lactose, and rely on flagellar movement. Growth is observed at 30 °C and 37 °C. Catalase activity is variable [4], along with lack of saccharolytic activity, and mostly are o-nitrophenyl-β-D-galactopyranoside (ONPG) negative [1, 2].

**Fig. 3:**
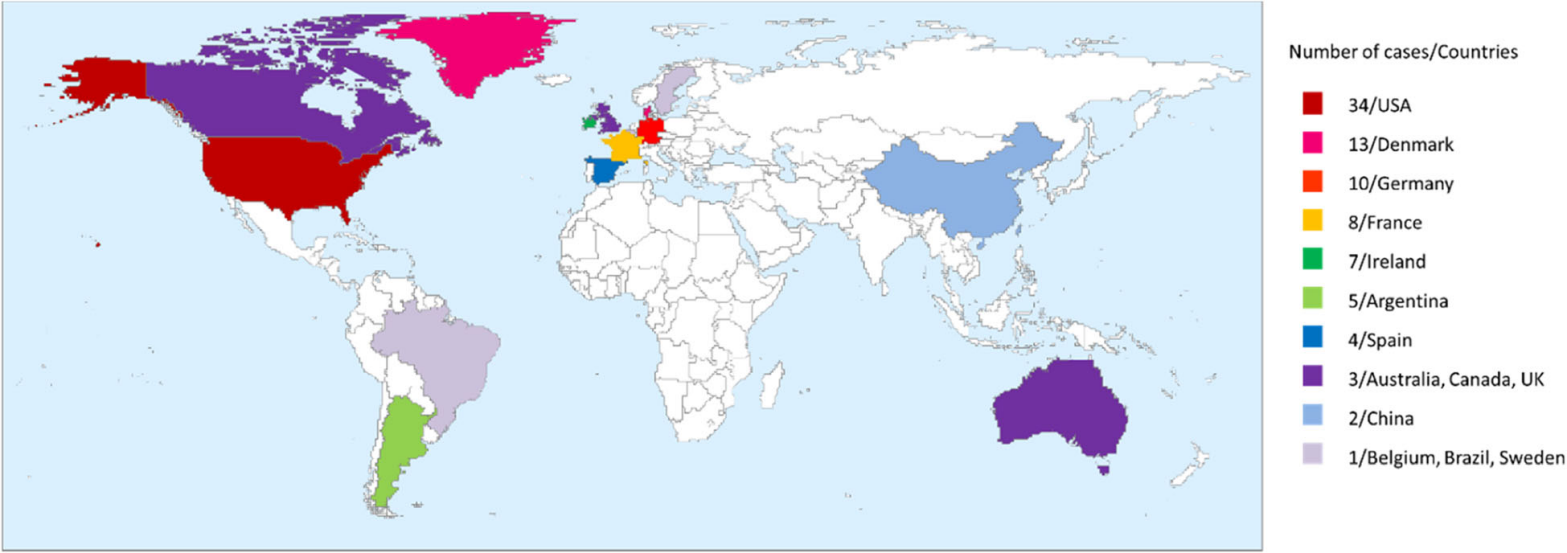
Global distribution of Pandoraea spp. Most *Pandoraea* cases occurred in Europe, America, and Australia, which was consistent with the epidemiology of CF

It presently comprises five species that have been isolated from human clinical specimens (*P. apista, P. pulmonicola, P. pnomenusa, P. sputorum,* and *P. norimbergensis*) [1], five named non-clinical species (*P. thiooxydans* [5]*, P. oxalativorans* [6]*, P. faecigallinarum* [6]*, P. vervacti* [6], and *P. terrae* [4]) that have been isolated only from non-clinical origin and at least four unnamed genomospecies [1, 7]. The main sources of *Pandoraea* species in the environment including soil, animal feces, water, and even powdered milk [8]. The clinical species were mainly isolated from respiratory specimens of cystic fibrosis (CF) patients [1, 3, 7, 9–24]. To date, cases of *Pandoraea* species caused colonization or infection have been reported all over the world, including USA [1, 7, 12, 25], Denmark [1, 10, 26], Germany [13], France [18, 19], Ireland [26], Argentina [20, 24], Spain [16, 17, 27, 28], Australia [3, 14, 15], Canada [1], UK [1, 9, 22], China [29], Belgium [1], Brazil [1], Sweden [1] (See at Fig. 3). Most cases occurred in Europe, America, and Australia, which was consistent with the epidemiology of CF [30].

From 1974 to 2014, only 34 cases of CF were reported in China [31]. *Pandoraea* species seem to be rare in non-CF patients, which may be the major reason that the first clinical isolate was reported not until the end of 2018 in China. But in our opinion, the incidence of infections caused by *Pandoraea* species is underestimated due to the difficulty in identification.

Identification of *Pandoraea* species through routine diagnostic laboratories, such as phenotypic methods and VITEK 2 automatic microorganisms analyzer, can commonly lead to misidentification [1, 7, 14, 15, 20, 29]. Molecular analysis for further confirmation is necessary when an isolate is unclearly identified. The cellular fatty acid analysis may be useful [1, 2], and yet cellular fatty acid-deficient isolate has been reported [15]. Genus-specific PCR assays and the sequences of 16S rRNA [1, 32] and gyrB [33] genes have proven to be reliable but may have some limitation in differentiating the *Pandoraea* species [15, 29]. More recently, several studies have reported good results in using MALDI-TOF MS for the identification of *Pandoraea* species [16–18, 20]. MALDI-TOF MS is a quick, easy and practical, high throughput analytic method that relies on a comparison between the mass spectrum of the isolate and the mass spectra in available databases. But *Pandoraea* species are rare in clinical isolates. With less information contributed by *Pandoraea* species in the database used might limit the discriminatory power of this method. Misidentification in strains-level has been reported [3]. MALDI-TOF MS is a promising approach, but more specific information is needed to update its database for accurate confirmation of bacteria that is less common. But with MALDI-TOF MS, quick identification, in the beginning, becomes possible. Martina and et al. [24] reported “the first case of *Pandoraea sputorum* colonization in Argentina” in 2017, but two *Pandoraea sputorum* strains, along with a *Pandoraea apista* and a *Pandoraea pulmonicola*, had been re-identified from 396 non-fermenting Gram-negative bacilli clinical isolates from a hospital in Argentina in 2015 [20]. Colonization and infection associated with *Pandoraea* species may have always existed, but were missed by the approach we used in the past.

### Pathogenicity

The pathogenicity of *Pandoraea* species in CF patients remains controversial. In many cases, a degree of deterioration has been observed after *Pandoraea* species were cultured together with some other bacteria in a respiratory specimen. But bacteremia caused by *Pandoraea* species in CF patients has been reported [11]. Some studies show that *Pandoraea* species may spread between CF patients [10, 19]. *Burkholderia* sp., which belongs to the same family as *Pandoraea* species, is generally considered transmissible and may cause severe infection after lung translation [34]. Many experts strongly recommend isolation for CF patients infected or colonized with *Burkholderia cepacia* [35]. Although the study of Pimentel et al. [14] shows that colonized with *Pandoraea* species before lung transplantation in CF patients may not be a predictor of poor outcomes after transplantation. It is important to find out the pathogenicity and transmissibility of *Pandoraea* sp. in CF patients.

Reports of non-CF patients infected or colonized with *Pandoraea* species are summarized in Table 2. Unlike CF patients, *Pandoraea* infection seemed to be more likely to cause bacteremia in non-CF patients [1, 7, 25, 27, 29], and co-presenting with other pathogens is only reported in two cases [25, 28]. In the study of Coenye et al. [1] and Daneshvar et al. [7], *Pandoraea* species cultured from respiratory specimens of non-CF patients have been reported, but with less case information. It makes our case the first well-reported case of pneumonia potentially caused by *Pandoraea* species in non-CF patients. Pneumonia was also reported in the case of Stryjewski et al. [25]. And yet, *Pandoraea pnomenusa* was cultured only from his blood samples, but not from respiratory samples. Furthermore, co-existing with nocardiosis and mycetomas makes it confusing to identify the responsible pathogen of pneumonia. It is reasonable to see *Pandoraea* species as an opportunistic pathogen in non-CF patients.

**Table 2:** Reports of non-CF patients infected or colonized with *Pandoraea* species^a^

Reference	Strains	Sources^b^	Age/sex/underlying illness	Location	Other pathogens^c^	Outcomes
Coenye 2000 [1]	*P. norimbergensis*	Blood	NG	Belgium	NG	NG
	*P. norimbergensis*	BALF	NG	Sweden	NG	NG
Daneshvar 2001 [7]	*P. apista*	Blood	66 yr./F/COPD	California,USA	NG	NG
	*P. apista*	BALF	75 yr./F/NG	California, USA	NG	NG
	*P. pnomenusa*	Blood	46 yr./M/NG	Texas,USA	NG	NG
	*Pandoraea* UG 2	MS	NG/F/NG	Georgia,USA	NG	NG
	*P. pnomenusa*	Blood	76 yr./M/NG	Hawaii,USA	NG	NG
	*P. pnomenusa*	Blood	49 yr./M/NG	Louisiana,USA	NG	NG
	*Pandoraea*	Sputum	71 yr./F/NG	Utah,USA	NG	NG
Stryjewski 2003 [25]	*P. pnomenusa*	Blood	30 yr./M/NC, MC	USA	*Nocardia sp.*	Died
Falces 2016 [27]	*P. pnomenusa*	Blood	10mth/NG/ALL	Spain	none	Alive
Monzón 2018 [28]	*P. sputorum*	HDC	79 yr./M/MM, ESRD, HP, T2DM	Spain	*E. coli,* *S. maltophila,A. baumannii,* *S. epidermidis*	Alive
GAO 2018 [29]	*Pandoraea sp.*	Blood	23 days/M/NJ	China	none	Alive
Our case	*P. apista*	sputum	44 yr./M/ MI, BT	China	none	Died

^a^ *BALF* bronchoalveolar lavage fluid, *MS* maxillary sinus, *HDC* hemodialysis catheter, *NG* no given, *yr*. years, *mth* months, *F* female, *M* male, *COPD* chronic obstructive pulmonary disease, *NC* nocardiosis, *MC* mycetomas, *ALL* acute lymphoblastic leukemia, *MM* multiple myeloma, *ESRD* end-stage renal disease, *HP* hypertension, *T2DM* type 2 diabetes mellitus, *NJ* neonatal jaundice, *MI* multiple injury, *BT* brain trauma, *USA* the United States of America

^b^ OSources of the strains

^c^ Other pathogens presented with *Pandoraea* sp

Some studies have investigated the pathogenesis of *Pandoraea* species. The ability to trigger a pronounced pro-inflammatory response, with an elevation of both interleukin (IL)-6 and IL-8 has been reported in the study of Caraher et al. [26]. This study also demonstrates that only a few strains have the abilities to invade lung epithelial cells (3 out of 19) and form biofilms (1 out of 19). According to Costello et al. [36], cellular invasion of *Pandoraea* species is independent of CF phenotype, and *Pandoraea* strains were also capable of translocation across polarized lung epithelial cell monolayers. The lack of enhanced susceptibility to the invasion of cells with a CF phenotype over non-CF cells is also discovered in their study. This may be one of the reasons that *Pandoraea* species more commonly lead to colonization rather than bacteremia in CF patients. Further research [37] shows that co-colonized with *Pseudomonas aeruginosa* may be another reason that *Pandoraea* species behave more gently in CF patients. *Pseudomonas aeruginosa* can inhibit the growth of *P. pulmonicola* and *P. apista* and the pro-inflammatory effects caused by these strains. These findings agree with a summary of *Pandoraea* species infections in transplant patients produced by Pimentel et al. [14], in which 5 cases of lung transplant patients have been reviewed and found out that CF patients previously colonized with *Pandoraea* species and *Pseudomonas aeruginosa* seem to have a better survival after transplantation than the only one non-CF patient, who was without *Pseudomonas aeruginosa* colonized previously and died after transplantation due to *Pandoraea pnomenusa* bacteremia.

Molecular biotechnology has been used to analyze virulence genes and drug resistance genes of *Pandoraea* species. Lim et al. [22] sequenced the complete genome of a *Pandoraea pnomenusa* strain and identified 16 virulence factors, which are well-characterized virulence determinants in some other pathogens. Robson et al. [38] sequenced the complete genome of two *Pandoraea pnomenusa* strains and found 130 gene sequences related to virulence, disease and drug resistance.

Mobile genetic elements (MGE), such as plasmids, can spread virulence and antibiotic resistance genes between microbes [39]. Yong et al. [21] analyzed one plasmid from *Pandoraea apista* and 7 plasmids from non-clinical *Pandoraea* strains (*Pandoraea faecigallinarum*, *Pandoraea thiooxydans*, and *Pandoraea vervacti*). More virulence genes were found in non-clinical strains than in *Pandoraea apista* and antibiotic resistance genes were only detected in the plasmids from non-clinical *Pandoraea* strains. This means that the *Pandoraea apista* strain analyzed is less virulent and lack of the ability to spread antibiotic resistance genes through plasmid. But with only one clinical strain detected, we do not know if this conclusion could be extended to all the clinical *Pandoraea* strains. However, many opportunistic pathogens are found transmit into clinical settings from the environment nowadays. These non-clinical strains may also be a thread.

### Susceptibility

In the study of Daneshvar et al. [7] in 2001, susceptibility result of some strains is not given individually. So we take the isolates from the same strain of *Pandoraea* as one isolate and using the mode minimum inhibitory concentration (MIC) to determine the susceptibility.

To dates, there are no breakpoints for the results of antimicrobial susceptibility tests suggested for *Pandoraea* species. Interpretive susceptibility criteria suggested for *Burkholderia cepacia* complex [19], other Non-*Enterobacteriaceae* [12, 17, 24], *Pseudomonas aeruginosa* [3, 29], or *Stenotrophomonas maltophilia* [3] was used to determine the results of susceptibility tests. Different criteria, and in some cases, different susceptibility methods might lead to unavoidable bias. Antimicrobial susceptibility profiles of *Pandoraea* species reported in the literature are summarized in Table 3. From Table 3, we can tell that *Pandoraea* is resistant to most antibiotic agents in most cases, such as aminoglycosides, most β-lactam agents and quinolones. However, the sensitivity of *Pandoraea* to piperacillin, piperacillin-tazobactam, aminoglycosides, and fluoroquinolones is variable. *Pandoraea sputorum* seems to be more sensitive to piperacillin-tazobactam than any other strains. *Pandoraea apista* is the only strain that shows sensitivity to ciprofloxacin and sparfloxacin in some studies. In contrast, most *Pandoraea* strains are sensitive to imipenem, tetracycline, and trimethoprim-sulfamethoxazole. It is important to note that almost all the *Pandoraea* isolates demonstrated resistance to meropenem but most of the strains are sensitive to imipenem, which is exactly on the opposite of *Burkholderia*. Even though they are closely related. Agents that have a potential activity to *Pandoraea* genus but fewer data reported include doxycycline [11], minocycline [27], tigecycline [18], and rifampicin [18, 19].

**Table 3:** Susceptibility profiles of *Pandoraea* species^a^

Reference	Strains	Methods^b^	Interpretive susceptibility criteria^c^	Drug(s) to which organism was:		
				Sensitive	Intermediate	Resistant
Daneshvar 2001 [7]	*P. apista*	BMD	NG	AMK,CIP,IMP,SPX,TET	CHL,TOB	AMP,AMC,CZO,CTX,FOX,GEN,MEM
	*P. pnomenusa*			IMP,SPX,TET	CHL	AMP,AMC,AMK,CZO,CTX,FOX,CIP,GEN,MEM,TOB
	*Pandoraea sp.*			IMP		AMP,AMC,AMK,CZO,CTX,FOX,CHL,CIP,GEN,MEM,SPX,TET,TOB
	*Pandoraea sp.*			IMP,SPX	TET	AMP,AMC,AMK,CZO,CTX,FOX,CHL,CIP,GEN,MEM,TOB
	*Pandoraea sp.*			none	CHL,TET	AMP,AMC,AMK,CZO,CTX,FOX,CIP,GEN,MEM,IMP,SPX,TOB
Moore 2002 [9]	*P. apista*	BMD	NG	TOB,TZP,IMP,CIP	none	GEN,CAZ,TEM,AZL,MEM,ATM,COL
Jørgensen 2003 [10]	*P. apista*	KB	PTM	TET,SMZ,SMT	CRO,CAZ,MEM,THI	aminoglycosides,most β-lactam (penicillin, AMP),quinolones (CIP,OFX,CFN),CHL,TMP,macrolides
Stryjewski 2003 [25]	*P. pnomenusa*	KB	NG	IMP	none	aminoglycosides, CAZ,CIP,TZP,SMT
Johnson 2004 [11]	*P. apista*	NG	NG	SMT (sputum)	IMP,DOX,CRO	AMK,ATM,FEP,CAZ,CIP,GEN,MEM,TZP,TIC,TOB,SMT (blood)
Atkinson 2006 [12]	*P. apista*	KB	ONE	CRO,SMT	none	AMP,GEN,TOB,IMP,CZX,TZP,COL,ATM
	*P. apista*			SMT,FEP,CRO,TZP	none	AMP,GEN,TOB,IMP,AMK,COL,ATM
Pimentel 2008 [14]	*P. sputorum*	KB	NG	PIP,TZP	none	NG
Martínez 2011 [16]	*P. sputorum*	E-test	NG	TZP,IMP,SMT	none	CAZ,FEP,ATM,MEM,TOB,AMK,COL
Fernández 2012 [17]	*P. sputorum*	BMD	ONE	TZP,IMP,SMT	none	AMX,AMC,CTX,CAZ,MEM,GEN,TOB,AMK,CIP,COL,AZM
Kokcha 2013 [18]	*P. pulmonicola*	NG	NG	TGC,RIP	none	TIC,TIM,CAZ,IMP,GEN,TOB,FOS,SMT,COL,CIP,CPO,FAR,MEM
Schneider 2006 [13]/2015 [40]	*P. pnomenusa*	KB	NG	TET	none	AMX,PIP,TZP,CAZ,CTX,FOX,ATM,MEM,IMP,FRO,GEN,TOB,CIP,SMT,CHL
	*P. pnomenusa*			IMP,TET	CTX	AMX,PIP,TZP,CAZ,FOX,ATM,MEM,GEN,TOB,CIP,SMT,CHL
	*P. pnomenusa*			IMP,SMT,TET	CTX	AMX,PIP,TZP,CAZ,FOX,ATM,MEM,GEN,TOB,CIP,CHL
	*P. apista*			IMP,SMT,TET	CTX	AMX,PIP,TZP,CAZ,FOX,ATM,MEM,GEN,TOB,CIP,CHL
	*P. norimbergensis*			IMP,SMT,TET	CTX,CHL	AMX,PIP,TZP,CAZ,FOX,ATM,MEM,GEN,TOB,CIP
	*P. pulmonicola*			IMP,SMT	CTX	AMX,PIP,TZP,CAZ,FOX,ATM,MEM,GEN,TOB,CIP,CHL,TET
	*P. sputorum*			IMP,SMT,TET	none	AMX,PIP,TZP,CAZ,CTX,FOX,ATM,MEM,GEN,TOB,CIP,CHL
	*P. sputorum*			IMP,SMT,TET	CTX	AMX,PIP,TZP,CAZ,FOX,ATM,MEM,GEN,TOB,CIP,CHL
	*Pandoraea sp.*			IMP,SMT	CTX	AMX,PIP,TZP,CAZ,FOX,ATM,MEM,GEN,TOB,CIP,CHL,TET
	*Pandoraea sp.*			IMP,SMT	CTX	AMX,PIP,TZP,CAZ,FOX,ATM,MEM,GEN,TOB,CIP,CHL,TET
Degand 2015 [19]	*P. pulmonicola*	NG	*B. cepacia* complex	SMT,RIP	none	PIP,TZP,CAZ,FEP,IMP,MEM,CIP,COL
Ambrose 2016 [3]	*P. pnomenusa*	KB	*P. aeruginosa*, *S. maltophilia*	IMP,SMT	none	CAZ,CIP,GEN,TOB,TZP,TIM,ATM,CRO,MEM,COL,TMP
Falces 2016 [27]	*P. pnomenusa*	NG	NG	MIN,IMP	none	NG
Martina 2017 [24]	*P. sputorum*	KB	ONE	SMT,IMP	none	NG
GAO 2018 [29]	*Pandoraea sp.*	KB	P. aeruginosa	IMP,TET,SMT,SAM	TZP	CAZ,AMK,ATM,GEN,TOB,PIP,FEP,CIP,LEV,MEM,TIC

^a^ *BMD* Broth microdilution, *KB* Kirby-Bauer test, *NG* not given, *PTM* provided by the manufacturer, *ONE* Other non-Enterobacteriaceae, *AMC* amoxicillin-clavulanic acid, *AMP* ampicillin, *AMX* amoxicillin, *ATM* aztreonam, *AZL* azlocillin, *AZM* azithromycin, *CAZ* ceftazidime, *CFN* clindamycin, *CHL* chloramphenicol, *CIP* ciprofloxacin, *COL* colistin, *CPO* cefpirome, *CRO* ceftriaxone, *CTX* cefotaxime, *CZO* cephazolin, *CZX* ceftizoxime, *FEP* cefepime, *FOS* fosfomycin, *FOX* cefoxitin, *FRO* faropenem, *GEN* gentamicin, *IPM* imipenem, *LEV* levofloxacin, *MEM* meropenem, *OFX* ofloxacin, *PIP* piperacillin, *RIP* rifampicin, *SAM* ampicillin-sulbactam, *SPX* sparfloxacin, *SMT* trimethoprim-sulfamethoxazole, *SMZ* sulfamethoxazole, *TEM* temocillin, *TET* tetracycline, *THI* thienamycin, *TIC* ticarcillin, *TIM* ticarcillin-clavulanate, *TMP* trimethoprim, *TOB* tobramycin, *TZP* piperacillin-tazobactam

^b^ Methods of antimicrobial susceptibility testing

^c^ Criteria used to determine the results of *Pandoraea* sp. susceptibility test

Enzyme-production is one of the most important mechanisms of bacterial resistance to antibacterial agents. Carbapenems are essential for some severe multi-drug resistant bacterial infections. Germs that can produce carbapenemases are seeing as a great threat to human beings [41]. Schneider et al. [13] found out that Oxacillinases-62 (OXA-62) is involved in the mechanism of resistance to imipenem. OXA-62 is only reported in *P. pnomenusa* species, and yet resistance to imipenem of *Pandoraea apista* [12] and *Pandoraea pulmonicola* [18, 19] have been reported, indicating that there may be more than one mechanism involved. The MICs of meropenem in this study was reduced by 8 times after adding efflux pump inhibitors, which indicating resistance of *P. pnomenusa* to meropenem may contribute by two mechanisms including producing OXA as well as an efflux pump. The researchers subsequently sequenced the oxacillinases of nine isolates belong to six *Pandoraea* species and found nine novel oxacillinase variants (OXA151-OXA159) [40]. 1All the strains are resistant to meropenem, but the MICs of meropenem can be reduced by 4–32 times by adding an active-site serine β-lactamases inhibitor, confirmed that these new oxacillinases also have the ability to hydrolyze meropenem.

*Pandoraea*. is a new genus that has been classified within 20 years. We report a case of a patient admitted to the ICU after removal of traumatic intracranial hematoma and decompressive craniectomy, who subsequently developed *Pandoraea* related pneumonia and eventually died of multiple organ failure. Through a literature review, we learned that *Pandoraea* sp. is a multi-drug resistant opportunistic pathogen, which can cause pneumonia and bacteremia by several mechanisms. Although this bacterium is more commonly found in CF patients, there have been reports of infection in non-CF patients, and there is evidence supporting *Pandoraea* species could be more virulent in non-CF patients. The genus is usually sensitive to imipenem, tetracycline, and SMT. However, the susceptibility is highly variable. Species-level identification and antibiotic susceptibility test are necessary.

## Data Availability

All the data and material involved in the current study are available from the corresponding author on reasonable request.

## Abbreviations

CF: Cystic fibrosis
COPD: Chronic obstructive pulmonary disease
CT: Computed tomography
EICU: Emergency intensive care unit
hsCRP: hypersensitive C-reactive protein
IL: Interleukin
MALDI-TOF MS: Matrix-assisted laser desorption ionization–time-of-flight mass spectrometry
MIC: Minimum inhibitory concentration
OXA: Oxacillinases
PCT: Procalcitonin
PFGE: Pulsed field gel electrophoresis
